# Potential traumatic events and symptoms of post-traumatic stress in unaccompanied refugee minors—a comparison with youth in foster care

**DOI:** 10.1007/s00787-021-01876-6

**Published:** 2021-09-19

**Authors:** Ingrid Kvestad, Tormod Bøe, Nawar Sayyad, Jens Christoffer Skogen, Sølve Randal, Stine Lehmann

**Affiliations:** 1 Regional Centre for Child and Youth Mental Health and Child Welfare, NORCE Norwegian Research Centre, Bergen, Norway; 2 Department of Psychosocial Science, Faculty of Psychology, University of Bergen, Bergen, Norway; 3 Child Welfare Services for Unaccompanied Refugee Minors, Bergen Municipality, Bergen, Norway; 4 Department of Health Promotion, Norwegian Institute of Public Health, Bergen, Norway; 5 Department of Public Health, Faculty of Health Sciences, University of Stavanger, Stavanger, Norway; 6 Alcohol and Drug Research Western Norway, Stavanger University Hospital, Stavanger, Norway; 7 Department of Health Promotion and development, Faculty of Psychology, University of Bergen, Bergen, Norway

**Keywords:** Unaccompanied refugee minors, Foster youth, Potential traumatic events, Post-traumatic stress, Child welfare services

## Abstract

Building knowledge on how child welfare services (CWS) should tailor services for unaccompanied refugee minors (URMs) is important. URMs and youth in foster care are high-risk groups taken care of by the CWS in Norway. Little is known on whether knowledge gained from youth in foster care can inform services for URMs, and if these groups are comparable in terms of experiences of potential traumatic events (PTEs) and post-traumatic stress disorder (PTSD) symptom load. Eighty-one URMs reported PTEs and PTSD-symptoms using an adapted version of the Child and Adolescent Trauma Screen (CATS). Responses were described and compared with a sample of 303 youth in foster care in linear regression models. We present relative risks (RR) and standardized mean differences (SMD) for the PTEs and the PTSD subscale and total score between the groups in forest plots. URMs had experienced a mean (standard deviation) of 6.4 (3.4) PTEs and 43.9% reported to have PTSD-symptoms at or above the clinical cut off. Compared to the foster youth, URMs reported more exposures of interpersonal violence outside of the family (RRs ranging from 66.4 [95%CI 18.1; 243.5) to 1.3 (1.0, 1.5)], and more PTSD-symptoms in the re-experiencing subscale [SMD = 0.3 (95% CI 0.1, 0.6)]. The frequency and types of PTEs and the PTSD-symptom load and profile among URMs and youth in foster care differed. Findings underscore the importance of qualified and targeted care for URMs, and that this care should differ to that of other high-risk groups in the CWS.

## Introduction

About half of the refugees in the world are less than 18 years and a large proportion of these are unaccompanied refugee minors (URMs) [1]. URMs refers to children under the age of 18 who have fled their country of origin without parents or other legal guardians to seek asylum in a new country [2]. In 2015 the rate of URMs arriving in European countries raised dramatically. In Norway, approximately 5000 URMs applied for asylum and was granted residence [3, 4]. The elevated number of URMs granted residence led to a mobilization of the Norwegian municipalities to increase the number of settlements. Despite the substantial decrease in the numbers of URMs seeking asylum seen after 2018 [4], it is important to utilize the experiences of the municipalities and the previously settled URMs to build knowledge on how to tailor services for URMs upon settlement in a new host country.

In Norway, the municipalities have the main responsibility for ensuring the health and well-being of their inhabitants. As in most high-income countries, it is mainly the child welfare services (CWS) that are responsible for placement and follow-ups for URMs such as access to education and health services [5, 6]. To enhance the psychosocial development for these young people upon arrival, care strategies based on available evidence is crucial [6, 7]. We have increasing knowledge regarding youth in foster care`s adverse care experiences prior to placement and their needs after placement [8–11]. Less is known, however, about whether our knowledge of foster children is generalizable to URMs to such a degree that it enables policymakers and service-providers to tailor services to meet these young people`s particular needs. Hence, further research is called for to establish more effective support systems for URMs in the municipalities [7, 12, 13].

Studies repeatedly show that exposure to potential traumatic events (PTEs) during childhood are potent predictors of mental health problems among both adolescents and adults [14–16]. Arriving in their host country, URMs are at increased risk of having experienced adversities both in their home country and during their flight [17–19]. Previous studies among URMs report a high prevalence of symptoms of post-traumatic stress disorder (PTSD), depression, and anxiety, with adolescents and females at particular risk for mental health problems [7, 12, 20]. Exposure to violence, separation from family members, death of parents and close relatives and exposure to armed conflicts are stressful life events frequently reported by the URMs before migration. A recent German study in URMs approximately 22 months after resettlement, demonstrates high levels of psychological distress, with almost 70% of the participants scoring above the clinical cut-off for PTSD [21]. Among these URMs, increased numbers of PTEs were the strongest predictor for psychological distress. Moreover, re-experiencing symptoms seemed to be the most prominent symptoms for these adolescents [22]. In a Norwegian longitudinal study following a cohort of 95 URMs settled in Norwegian municipalities over a five-year period, the initial high levels of depression decreased significantly after 5 years, while anxiety, PTSD- and externalizing symptoms remained high [23–25]. These results, underline the need for not only short-term follow-up of mental health problems for URMs, but also close follow-ups on long-term after settlement [24].

In Norway, URMs and youth in foster care are both considered high-risk groups under the care of the CWS. While the CWS is responsible for timely screening, access to treatment and tailored follow-ups for both, the work with these two high risk groups is primarily separated in the agencies, with specific institutions, group homes and foster families designated for the URMs. In a recent study, youth in foster care reported exposure to an average of more than three PTEs, and 53% had PTSD-symptoms at or above clinical cut-off [9]. Moreover, exposure to increased numbers of PTEs was associated with an increased PTSD-symptom load. There is an identified lack of systematic knowledge on how the CWS can contribute to a healthy development for URMs after settlement in Norway [5, 26]. To date, little is known about to which extent URMs and youth in foster care as groups are comparable in terms of their exposure-profiles and symptom-load and patterns. In other words, whether the knowledge base gained from youth in foster care could inform services for the URMs, needs further examination. Therefore, the objectives of the current study were to describe the frequency and types of exposure to PTEs in URMs and compare their exposure-profile with that of youth in foster care. Second, to describe the PTSD-symptoms load and profile among URMs and compare these to those of youth in foster care. We believe that such comparisons may provide important information on how services should be tailored for URMs upon arrival and help identify interventions specifically targeted to enhance long-term outcomes of URMs.

## Methods

### Study sample

Data for the current study were collected in the Pathways to Independence study, a comprehensive survey conducted among URMs settled in Bergen Municipality, Norway [27]. All participants were granted residence permit in Norway and were cared for by the CWS for URMs in the municipality (URMs CWS). The URM CWS has the sole responsibility for settlements of URMs in the municipality of Bergen. Their services include both case work and placements in foster homes, institutions, joint homes, host families and private housing with or without follow-up.

The URM CWS coordinated the data collection for the study. Data collection lasted from December 2018 through January 2019, and we invited all URMs who were 15 years or older and who consented to participate in the study. For participants younger than 16, consent was also obtained from their legal guardian. From the target population of 116 URMs, ten URMs were considered ineligible to participate due to evasive behavior and poor mental health, three were excluded due to inability of the URMs CWS to provide the participants with proper follow-up after the survey (i.e., participants living in other parts of the country), and two were excluded due to that the case worker was not available. Hence, the number of invited URMs was 101 of which 81 consented and were included, yielding a participation rate of 80%. Participant`s country of origin, age and sex were representative for this group of adolescence in Norway [3].

The study was approved by the Regional Committee for Medical and Health Research Ethics of Western Norway (2018/966) and conducted in accordance with recommendations from the Norwegian Data Protection Services. The participation in the study was voluntarily, and the participants could withdraw from the study at any time.

### Procedures

The case workers at the URMs CWS were responsible for inviting the URMs to participate in the study, and to schedule a time to complete the survey. The comprehensive survey was developed particularly for this study and included questions on background information and satisfaction with the URMs CWS in addition to a range of standardized and validated questionnaires measuring exposure to potential traumatic events, symptoms of mental health problems and somatic complaints, sleep patterns and problems, protective factors, and quality of life [27]. The URMs completed the online survey at the case worker`s office. They were first provided with written and oral information about the study and consented to participate on the first page of the online questionnaire after going through the information thoroughly with the case workers. The case workers were present and available for questions and queries while the URMs filled in the survey but were instructed not to see the participants’ responses. The questions were not translated to the native languages of the URMs, but the case worker discussed with each participant whether he/she needed a translator when filling in the survey. Six of the participants used a translator due to lack of sufficient knowledge in the Norwegian language. When there was no translator, the case workers clarified words and sentences that the URMs found difficult to understand. All participants received a gift certificate of 300 NOK (approx. €30) as a compensation for their participation.

### Comparison group

The comparison group is derived from a study of youth in foster care (*N* = 302, 41.9% response rate), aged 11–17 years [mean (SD) 14.8 (2.05)] and 53% were male. Participants had lived with their current foster family for a mean (SD) duration of 6.7 (4.36) years. Data collection was completed from October 2016 through March 2017. Young people in foster care born between 1999 and 2005 whom had lived in their current foster placement for at least six months following legally mandated placement were deemed eligible. Eligible youths were identified through regional registers and through contact with municipal CWS and were invited through postal letters. Participants completed the survey online and were compensated with a gift card of 300 NOK (approx. €30). Details on procedure and recruitment are described elsewhere [9].

The study was approved by the Regional Committee for Medical and Health Research.

Ethics of Western Norway (2010/2367-1). Before combining data from the two studies, anonymization methods were employed, and the data were converted into aggregated statistical format for secondary analyses. The procedure was vetted by the data protection officer of the project-managing organization.

### Measures

To measure PTEs and PTSD we used the same instruments as in the study of youth in foster care [9]. These instruments were an adapted version of the Child and Adolescent Trauma Screen (CATS), translated to Norwegian by the Norwegian Center for Violence and Traumatic Stress studies. Part 1 of the CATS assess potential traumatic events (PTEs) that adolescence may experience, and part 2 cover symptoms of post-traumatic stress related to the PTEs in part 1.

#### Potential traumatic events (PTEs)

The PTEs included in the study were adapted from CATS part 1 and covered areas of interpersonal violence outside of the family, abuse within the family and sexual abuse. In the youth in foster care study, three items covering emotional neglect, physical neglect and emotional abuse, and two items on parentification due to neglect were added to the original 15 PTEs from the CATS part 1, while two items on experiencing serious disaster like a flood, tornado, hurricane, earthquake or fire and experiencing very scary events at the doctor, dentist or at hospital were left out from [9]. The list of the 18 PTEs was introduced in the questionnaire with the following instruction: “Below is a list of events children and young people may experience. If this happened to you, and you felt scared, confused or helpless, then mark Yes. If there are any questions you do not want to answer, mark Pass”. For each PTE, the participants scored No (0), Yes (1) or Pass (coded as missing), and hence the total possible score range from 0 to 18.

#### Symptoms of post-traumatic stress disorder (PTSD)

The questionnaire includes 20 items on symptoms of PTSD based on the DSM-5 criteria. The items cover the core symptoms of PTSD: Intrusion, avoidance, negative alterations in cognition and mood, and hyper-arousal [28]. Each question is scored “Never” (0), “Once in a while” (1), “Half of the time” (2) and “Almost always” (3) providing a total score ranging from 0 to 60. Scores between 15 and 20 indicates moderate trauma-related stress, and scores of 21 and more a probable PTSD-diagnosis. The questionnaire also includes five functional impairment items measuring the impact of the symptoms on everyday life such as getting along with others, hobbies, family relationships, school or work and general happiness. Each of these questions are scored “yes” (1) or “no” (0). Among US, German and Norwegian children, the CATS has demonstrated good to excellent reliability, satisfactory convergent-discriminant validity, and the data supported the underlying DSM-5 factor structure with four symptom clusters [31]. A recent study on Swedish youth aged 13–17 years, demonstrated good internal consistency on all four subscales, and the four-factor model for PTSD indicated good fit, reliability, and convergent validity. Here, CATS also discriminated between the nonclinical and clinical groups [29]. In the current study, the alpha-value for the total score was 0.93, ranging from 0.74 to 0.89 for the subscale scores indicating acceptable internal consistency.

### Statistical analyses

The difference in exposure to potential traumatic experiences (PTEs) between the two samples were investigated using a series of general linear models (GLMs) predicting exposure from a dummy variable indicating whether participants originated from the youth in foster care sample (coded as 0 and used as reference) or from the URMs sample (coded as 1). All GLMs were fitted using the binomial family with a log link, and estimates were subsequently exponentiated to obtain risk ratios and 95% confidence intervals (CIs) indicating the risk of exposure relative to the reference group of foster youth. Differences in symptoms of PTSD were investigated using the difference in the standardized mean response to each item by participants in the sample of URMs and the youth in foster care. The results are presented as a forest plot created with the R package “meta” [30]. The pooled overall and subscale effects for PTEs were created by manually calculating the standardized difference (Hedges’ g) between the pooled means of items using the R package “effsize” [31]. The same approach was used to investigate between-samples differences in scores on the CATS impact items. To visualize the distribution of symptoms in the two samples, a total symptom score was computed and is presented as a ridge plot [32]. One item from the CATS (enquiring about sleep difficulties) was by mistake excluded from the survey to the unaccompanied minor refugee asylum seekers, thus all analyses, including the calculation of the total symptom score, is based on the 19 items available in both samples. The distribution of age was different in the sample of youth in foster care (range = 11–18 years) relative to the URMs (range = 15–20 years). Sensitivity analyses adjusting for age did not yield substantively different results, and we therefore report results from unadjusted models. Data preparations and analyses were conducted with packages and functions from “tidyverse” [33] in R for Windows (version 3.6.3; R Core Team, 2020).

## Results

Demographic information of the study participants is provided in Table 1. Of the 81 URMs, more than 80% of the participants were males, the most frequent country of origin was Afghanistan (47%), followed by Eritrea, Syria, and Somalia. The mean [standard deviation (SD)] age of the participants was 18 (1.3) and the age ranged from 15 to 20 years. The most common living arrangements at the time of the study were private housing alternatives (40.2%) and host families/unattended dormitories (17.3%). While approximately 10% had not moved after settlement, approximately 40% reported to have moved 1 time, 40% 2–3 times and 8.6% to have moved 4–7 times.

**Table 1:** Demographic information of 81 unaccompanied refugee minors settled in a Norwegian municipality

	*N* (%)
Gender, boys	67 (82.7)
Age, years, m (SD)	18 (1.3)
15–16	10 (12.4)
17–18	38 (46.9)
19–20	33 (40.8)
Years in Norway (host country), m (SD)	3.5 (2.2)
Living arrangements	
Foster home	8 (9.9)
Joint homes	13 (16.1)
Institution	8 (9.9)
Host family or unattended dormitories	17 (17.3)
Private housing	35 (43.2)
Number of placements after settlement	
None	11 (13.6)
1	31 (38.3)
2–3	32 (39.5)
4–7	7 (8.6)
Country of origin	
Afghanistan	38 (46.9)
Eritrea	14 (17.3)
Syria	14 (17.3)
Somalia	8 (9.9)
Others	7 (8.6)
Number of PTEs, m (SD)	6.35 (3.4)
1–2	14 (18.2)
3–4	11 (14.3)
5–6	13 (16.9)
7–8	19 (24.7)
9–10	11 (14.3)
11 +	9 (11.7)
Moderate trauma-related stress, 15–20	7 (8.5)
Probable PTSD, 21–57	29 (35.4)

The URMs had experienced a mean (SD) of 6.4 (3.4) and a median (IQR) of 7 (3–9) PTEs each, ranging from 1 to 16. Table 2 shows the number of URMs that have experienced each PTE. Interpersonal violence outside of the family were the most frequent PTEs experienced by the URMs; 78% answered that they had experienced serious injury, sickness or sudden death of loved ones, 77% had experienced terror and war, 71% had witnessed others outside family being hit, kicked, pulled, injured, threatened or attack each other, and 63% had themselves been hit, kicked, pulled, injured or threatened by someone outside the family.

**Table 2:** Number (percentage) of potential traumatic events experienced by 81 unaccompanied refugee minors settled in a Norwegian municipality

	Yes
	*N* (%)
Interpersonal violence outside of the family	
Ever been involved in a serious accident	28 (37%)
Ever experienced terror or war	53 (77%)
Ever experienced serious injury, sickness or sudden death of loved ones	54 (78%)
Ever experienced bullying or threats	22 (32%)
Ever experienced abduction/kidnapping	18 (25%)
Ever been hit, kicked, pulled, injured or threatened by someone outside family	45 (63%)
Ever witnessed others outside family being hit, kicked, pulled, injured, threatened or attack each other	53 (71%)
Abuse	
Ever witnessed parent or other grown up in your home being hit, kicked pulled, injured, threatened or attack each other	27 (37%)
Ever been hit, kicked, pulled, injured or threatened by a parent or other grown up in your home	28 (38%)
Ever experienced a parent or other grown up in your home sweared at, offended, threatened, ridiculed, or being hurtful towards you	20 (31%)
Neglect	
Often cared for your own parents because they were unable themselves	18 (25%)
Often felt that your family did not love you—or that your family did not take care of you or each other	12 (17%)
Often felt that you did not have enough to eat, or you had to wear dirty clothes	26 (38%)
Often cared for your siblings because your parents were unable to	20 (28%)
Sexual abuse	
Ever someone taken pictures of your private body parts	< 5
Ever experienced someone touching, or yourself being forced to touch other private body parts	12 (17%)
Ever experienced rape (anal, oral, or vagal)	7 (9.3%)
Experienced anything else that made you feel confused or helpless	41 (64%)

**Fig. 1:**
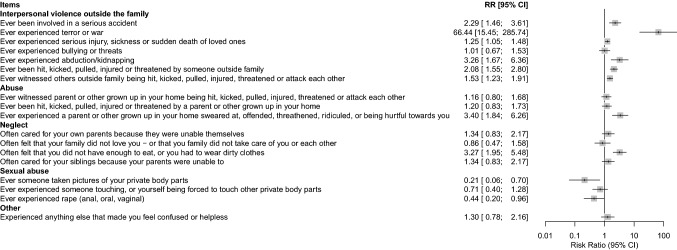
Forest plots indicating the risk of exposure to potential traumatic events in the unaccompanied refugee minors relative to the youth in foster care. Generated by general linear models, fitted by the binomial family with a logit link. The figure illustrates the relative-risk difference between youth in foster care sample (reference, shown as the vertical line at zero) and the unaccompanied minors (gray boxes). Boxes to the right of the vertical line indicate items where unaccompanied minors have higher relative risk than foster children, boxes to the left indicate items where unaccompanied minors have lower relative risk. *CI* confidence interval, *RR* risk ratio

Figure 1 shows the risk ratio for the URMs to have experienced the different PTEs, compared to the youth in foster care. Compared to the foster care sample, URMs more frequently reported to have experienced interpersonal violence outside the family such as terror and war, abduction/kidnapping, and been involved in a serious accident. They also reported more experiences of family violence. Moreover, the URMs more frequently reported exposure to physical neglect. Compared to the youth in foster care, the URMs reported less frequently sexual abuse such as rape and that someone had taken picture of their private body parts.

**Fig. 2:**
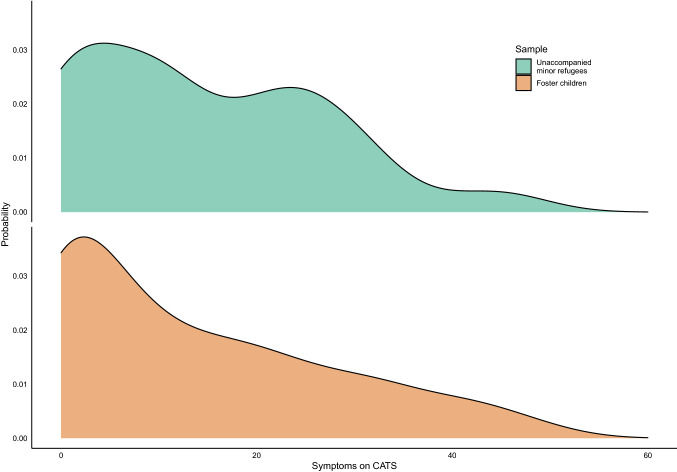
Density plots of the probability distribution of post-traumatic stress disorder among the unaccompanied minors and the youth in foster care. The figure shows the probability distribution of PTSD-symptoms among the URMs (top) compared to that of youth in foster care (bottom). The probability distribution (y-axis) indicates that URMs have a higher likelihood of symptom loads between 20 and 35 on CATS (x-axis), compared to foster children

Of the URMs, 8.5% had scores corresponding to moderate trauma-related stress and 35.4% had scores indicating a probable PTSD. Figure 2 shows the probability distribution of PTSD-symptoms among the URMs compared to that of youth in foster care. The distribution indicates that URMs have a higher likelihood of symptom loads between 20 and 35 on CATS, compared to foster children.

**Fig. 3:**
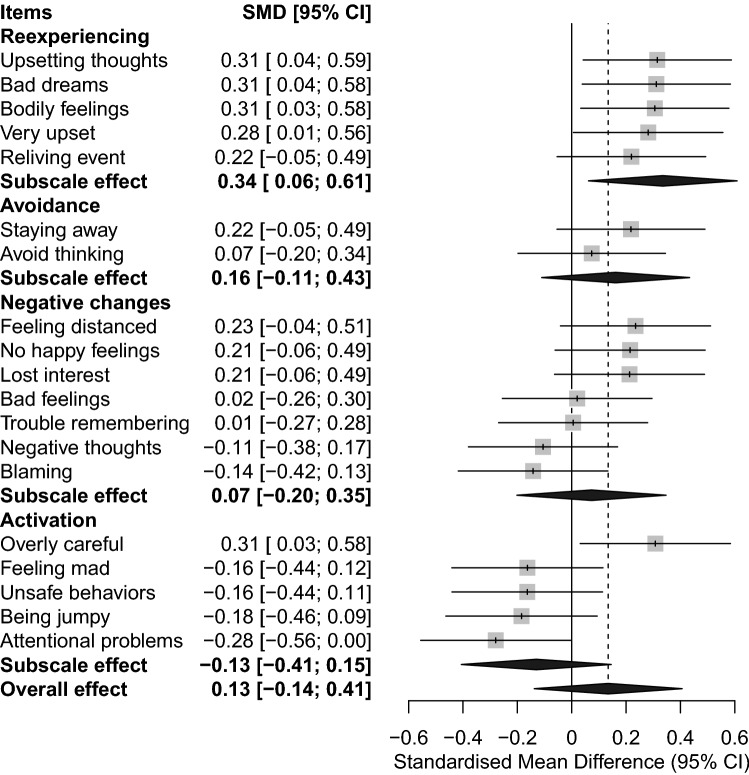
Forest plots indicating the differences in symptoms of post-traumatic stress (itemized, subscale and overall effect) in the unaccompanied minors and youth in foster care. The figure illustrates the standardized mean differences (SMD) on PTSD-items between foster children (reference, vertical line at zero) and unaccompanied minors (gray boxes). Boxes to the right of the vertical line indicate items where unaccompanied minors have a higher standardized mean than foster children, boxes to the left indicate items where unaccompanied minors a have lower standardized mean. The black diamonds are the SMD pooled over all items in each core PTSD-symptom domain. *CI* confidence interval, *SMD* standardized mean difference

Figure 3 shows the PTSD total- and subscale scores in the URMs compared to the foster care youth. While the URMs have higher scores on the re-experiencing subscale, there are no significant differences in scores on the other subscales or on the overall score between the two groups. URMs scored somewhat higher on the CATS impact items (mean score of 2.2) compared to the sample of foster children (mean score of 1.9), but the effect size was very small (Hedges *g* = 0.2, 95% CI [− 0.2, 0.5]) and non-significant.

## Discussion

In the current study, we have examined frequency and types of exposure to PTEs, and load and patterns of PTSD-symptoms reported by URMs cared for by the Norwegian CWS. We compared their responses with those of a group of youth in foster care, and our findings suggest that these groups differ in the frequency and profile of both exposure to PTEs and PTSD-symptoms. To our knowledge, this is the first study comparing the exposure and symptom profiles of URMs to that of foster youth, both high risk groups hard to recruit for research purpose. The uniqueness of the study is underscored by the fact that the two studies used the same assessment methods facilitating comparisons across these groups, which we believe provide meaningful knowledge for the tailoring of services to URMs in terms of targeted assessments and interventions.

### Comparisons of PTEs

The average number of 6.4 PTEs in the URMs is considerably above what was shown among youth in foster care, reporting on average 3.4 PTEs [9]. However, the range is somewhat similar for the two groups, with PTEs ranging from 1 to 16 among the URMs, and from 0 to 15 among youth in foster care*.* The reported number of PTEs in both these groups is considerably above what has been reported in a Norwegian community sample [34]. Comparing the exposure profiles between the URMs and the youth in foster care reveals clinically relevant differences between the groups. Findings from the foster care study show that these youth have been exposed to multiple types of interpersonal trauma [9]. The fact that the URMs report to have experienced more interpersonal violence outside of their families in terms of war, abduction and being witness to violence and death is not surprising considering their refugee status. Increased exposure to these traumas has also been confirmed in previous studies in URMs [20, 21, 23]. Our results indicate however, that URMs as a group carry a considerable heavier burden in terms of exposure to a greater load of different PTEs compared to youth in Norway in general, and to youth in foster care. Given that increasing numbers of PTEs is a verified risk factor for mental health problems [14], our findings underscore the importance of qualified and targeted care for URMs upon settlement, and moreover that this care should differ to that of other high-risk groups in the CWS-system.

Although not the most frequently reported traumatic experiences, it is important to note that across gender almost 10% of the URMs sample reported to have experienced rape and 17% reported being forcefully touched or being forced themselves to touch others` private body parts which is a clinically significant finding. Still, compared to the foster care sample, the URMs report less sexual abuse. This could be related to gender differences in the groups since there are few girls in the URMs cohort (14 of 81) and almost an even distribution among the foster care youth. In general, girls more frequently report to be exposed to sexual abuse then boys [35]. Our finding of relatively fewer reports of sexual abuse among URMs have been confirmed by others [21, 23]. In one of these studies, they found that sexual abuse was more common among female URMs (17%) than among males (9%) [23].

Comparing the magnitude of exposure in the current study to that of URMs in other studies is challenging given that the number of PTEs reported will differ according to assessment methods. A recent German study has also assessed PTEs by use of the CATS among a group of unaccompanied and accompanied refugee minors, reporting an average of almost nine PTEs [21]. Although using the same assessment tool, this study added items measuring migration-related events to the questionnaires, which could explain some of the differences in prevalence. Similarly, in the current study, items on physical and emotional neglect, emotional abuse, and parentification were added [9] making the comparisons with the German study even more complicated. Nevertheless, although challenging to compare, our findings confirm previous findings suggesting an increased risk for PTEs in URMs across countries of settlement [19, 20].

### Comparisons of PTSD symptoms

The proportion of URMs reporting symptoms at or above the clinical cut off for PTSD was 43.5% which is below the proportion demonstrated in the youth in foster care study where 52.9% reported symptoms at or above the clinical cut off [9]. Direct comparison of the responses between these groups shows however, that there are no significant differences in mean total- and subscale scores, except for in the re-experiencing subscale where the mean score was higher for the URMs. Our results indicate that among youth in foster care, the distribution of symptoms is more polarized between low-and high scores, whereas, for URMs there is an overall higher sub-clinical symptom load. Also, URMs seem to express post traumatic reactions through symptoms such as upsetting thoughts, bad dreams, and bad bodily feelings to a higher degree than youth in foster care. This finding of a particular symptom profile with relative heightened re-experiencing subscale is also found in other studies in URMs [22], suggesting that re-experiencing symptoms could be particularly salient in the refugee minor PTSD-symptomatology.

There is an identified lack of evidence-based interventions for URMs after settlement in their host country [13, 36]. Recent work on a trauma-focused group-based intervention led by trained and supervised child welfare staff show promising results for young refugees with sub-clinical PTSD-symptoms [37, 38]. There are also currently several studies that examine the effect of more comprehensive trauma-focused cognitive behavioral therapy (TF-CBT) in refugee minors who meet the diagnostic criteria of PTSD [39, 40]. Facilitating treatment in line with the individual symptom profiles of URMs, a stepped-care approach involving initial screening and subsequent allocation to a preventive group-based intervention or individual TF-CBT are currently being implemented and evaluated among young refugee minors in Germany [41].

Compared to other studies in URMs the prevalence of PTSD-symptoms seems low in the current study [20, 21, 23]. It is important to note that due to the unintended omission of an item on difficulties with sleep in the current study, direct comparisons of results with other studies are challenging. This sleep-item has shown to be of importance in understanding PTSD symptoms in URMs in previous studies using the CATS [22]. The etiology of mental health in URMs is complex and needs to be understood and addressed across multiple sectors that could potentially influence their mental health [42]. Hence, several mechanisms may explain the differences in symptom load across studies, such as differences in the level of exposure, time since leaving country of origin, perceived social support, level of support accommodation provided in the host country and access to targeted intervention, as well as individual traits such as cultural competence and the larger policy and political context [12, 19, 42].

Previous studies demonstrate that when the URMs are followed over time, although with some decline, the prevalence of PTSD-symptoms remains high [24, 25, 43]. Purgato and colleagues (2017) point to the fact that in the field of refugee mental health the consideration of other predictive variables than conflict-related traumatic events are called for [42]. A systematic review shows for instance that supportive living arrangements are important to promote well-being and improve mental health for URMs [13], with one study demonstrating that lower social support from mentors increase the risk of prolonged symptoms of PTSD [44]. In line with this, high degree of satisfaction with the follow-ups from the URMs CWS reported by the participants in our study [45] could be one mechanism that explain the comparably low prevalence of PTSD-symptoms. Taken together our findings underscore the importance of further research to fully understand how to secure long-term positive outcomes for this vulnerable group of refugee minors.

### Strengths and limitations

Major strengths of this study are the inclusion of two high-risk groups under the care of the CWS who are hard to recruit for research purpose, and the use of similar, validated questionnaires facilitating comparisons between these groups and placing our result into a relevant context for the CWS-system. A limitation to the study is that one of the 20 items in the CATS part 2 concerning PTSD symptoms was omitted. This limitation must be taken into consideration when comparing the findings to other investigations using the CATS but was accounted for in the current study by restricting the analysis to items completed in both samples. The distribution of age and gender differed between the samples, which represent another limitation to the study. Still post hoc sensitivity analyses adjusting for age did not yield substantively different results. The active role of the case worker in aiding the completion of questionnaires with the URMs could be viewed as both a strength and a limitation to the study. While the active role of the case workers could represent safety for the URMs, we cannot rule out that the responses of the URMs have been influenced by the fact that their case worker was sitting across the table. The aid of case workers was important to include the URMs to the study, still, a limitation of our study is the low sample size. We argue however, that a response rate of 80% and the comparable demographic information in terms of country of origin, sex and age with URMs on a country level [3] increase the generalizability of our results. The questionnaires were not translated to the URMs native language, and we cannot rule out significant language and cultural barriers for the participants in filling in the questionnaires. All participants were offered to use a translator, but only six accepted. Although most URMs did not feel a translator was necessary, we cannot rule out that the differences in scoring to that of the youth in foster care were due to understanding the questions and concepts differently. Moreover, one could argue that the measure of PTEs lacks nuances and is limited by its categorically scoring. Hence, only direct experiences with the PTEs are accounted for and not proximities that could also represent a threat for the individual. A multi-informant design, may have yielded a more nuanced picture of the conditions of the URMs [46], still information on traumatic events in early childhood would be challenging to assess given their situation. Another limitation to the current study is the cross sectional design with no follow-up data and hence, our inability to study trajectories of mental health in our participants. And finally, PTSD is only one of many relevant mental health outcomes of exposure to traumatic events, and a wider range of psychopathology especially symptoms of depression and anxiety could have given a broader insight into the situation of the URMs and the differences between these high-risk groups.

## Conclusion

Our findings demonstrate the magnitude of exposure to PTEs in URMs settling in a new country, the high degree of sub-clinical and clinical PTSD symptom load and the differences in exposure and PTSD-symptom profiles to that of youth placed in foster homes. Early screening and identification are important measures to secure tailored interventions targeting the specific experience and needs of URMs that deviate from other high-risk groups in care of the CWS. The comparably higher symptom load in the re-experiencing subscale suggest intervention targeting symptoms such as upsetting thoughts, bad dreams and bad bodily feelings are called for. Most URMs in Norway are placed in foster homes, institutions, joint homes, and host families. Educative measures to increase the competency on common sequala after exposure to PTEs, and how to support and care for youth struggling with PTSD symptoms, should be provided to both professionals and foster careers responsible for the daily care of URMs. As caring for trauma-exposed children may be challenging, and PTSD symptoms may be difficult to observe and understand, the CWS should be attentive to the need for support and guidance to careers of the young people. More knowledge is needed however, on how to secure long-term positive outcomes for URMs upon settlement in their host country.

## Data Availability

Upon request.

## Code availability

STATA 16.1 (StataCorp, College Station, Texas, USA), R version 3.5 for Mac (R Core Team, 2018).
